# Molluscum Contagiosum in a Pediatric American Indian Population: Incidence and Risk Factors

**DOI:** 10.1371/journal.pone.0103419

**Published:** 2014-07-29

**Authors:** Andrea M. McCollum, Robert C. Holman, Christine M. Hughes, Jason M. Mehal, Arianne M. Folkema, John T. Redd, James E. Cheek, Inger K. Damon, Mary G. Reynolds

**Affiliations:** 1 Division of High-Consequence Pathogens and Pathology, National Center for Emerging and Zoonotic Infectious Diseases, Centers for Disease Control and Prevention, Atlanta, Georgia, United States of America; 2 Epidemic Intelligence Service, Scientific Education and Professional Development Program Office, US Centers for Disease Control and Prevention, Atlanta, Georgia, United States of America; 3 Division of Epidemiology and Disease Prevention, Office of Public Health Support, Indian Health Service, Albuquerque, New Mexico, United States of America; University of North Carolina School of Medicine, United States of America

## Abstract

**Background:**

Molluscum contagiosum virus (MCV) causes an innocuous yet persistent skin infection in immunocompetent individuals and is spread by contact with lesions. Studies point to atopic dermatitis (AD) as a risk factor for MCV infection; however, there are no longitudinal studies that have evaluated this hypothesis.

**Methods:**

Outpatient visit data from fiscal years 2001–2009 for American Indian and Alaska Native (AI/AN) children were examined to describe the incidence of molluscum contagiosum (MC). We conducted a case-control study of patients <5 years old at an Indian Health Service (IHS) clinic to evaluate dermatological risk factors for infection.

**Results:**

The incidence rate for MC in children <5 years old was highest in the West and East regions. MC cases were more likely to have a prior or co-occurring diagnosis of eczema, eczema or dermatitis, impetigo, and scabies (p<0.05) compared to controls; 51.4% of MC cases had a prior or co-occurring diagnosis of eczema or dermatitis.

**Conclusions:**

The present study is the first demonstration of an association between AD and MC using a case-control study design. It is unknown if the concurrent high incidence of eczema and MC is related, and this association deserves further investigation.

## Introduction

Molluscum contagiosum (MC) is a skin disease caused by molluscum contagiosum virus (MCV), a poxvirus. The infection causes firm, small, papular lesions, often with an umbilicated center. Lesions may have a region of erythema and can be found anywhere on the body. Lesions may remain for many weeks to months before resolving without treatment; however, in immunocompromised individuals, infection with MCV can cause giant lesions and a large number of lesions that require treatment. Diagnosis is usually made upon visual inspection of the lesions. Although rarely pursued, laboratory confirmation is available. There are several approaches to treating MC lesions, including physical destruction (cryotherapy, curettage, laser therapy) or use of immunomodulatory medicines (topical imiquimod) [1], [2].

MCV is spread by contact with lesion material from an affected individual. Recommendations to prevent the spread of MCV on an individual's body or to others may include keeping the lesions covered and not sharing clothing, linens, or items that may contain lesion exudate with others [3]. Also, avoiding any behavior that would open the lesion and spread the material, such as shaving [4], scratching, and electrolysis [5], is recommended to prevent spread of the virus. An association between swimming pool use and MCV infection has been reported [6],[7], but robust scientific data do not exist to firmly support this association. Although benign, MC can have an adverse effect on patients' and their families' lives, causing emotional concern and anxiety about physical manifestations of the infection [8].

There have been notable clinical cases of patients who present with atopic dermatitis (AD, including eczema) and have concurrent extensive MC lesions [9], [10]. One clinical review of 284 children noted more lesions and a greater extent of surrounding eczema in patients presenting with MC and AD compared to those patients presenting with MC alone [11]. Indeed, there are indications that AD may be a risk factor for acquisition of MCV and that patients with AD may suffer a more extensive course of MC illness [12], but, to date, there are no case-control studies to evaluate these hypotheses.

Although MCV infection occurs in individuals of all ages, the infection is seen most often in children [13]–[15]. Publications often associate MC with tropical environments, the developing world, and poor living conditions, but infections are seen worldwide in a multitude of climates [8], [13], [16], [17]. An analysis of Indian Health Service (IHS) outpatient visit data showed a higher incidence of MC in American Indian/Alaska Native (AI/AN) children aged 1–4 years old compared to other age groups, and this rate was particularly high in the IHS West region [15]. This increased incidence and disparity seen among IHS AI/AN patients, specifically those in the West region, warranted further investigation of cases and their potential risk factors.

We present a study designed to determine potential dermatological risk factors of MCV infection among AI/AN children seen in IHS facilities with a high incidence of MC. Furthermore, we investigated the occurrence of those risk factors in patients with MC.

## Materials and Methods

### Incidence of MC at IHS outpatient facilities

The national Indian Health Service (IHS) direct and contract care outpatient visit data for fiscal years 2001–2009 were obtained from the IHS National Patient Information Reporting System (NPIRS) [18]. These data consist of all outpatient visit records from IHS-operated, tribally-operated and community hospitals and facilities that are contracted with the IHS or with tribes to provide healthcare services to eligible AI/AN persons [19]. The IHS administrative areas were classified into regions as described by the IHS: Alaska, East (Nashville), Northern Plains East (Bemidji), Northern Plains West (Aberdeen and Billings), Southern Plains (Oklahoma), Southwest (Albuquerque, Navajo, Phoenix, and Tucson), and West (California and Portland). Outpatient visit records were selected for AI/AN children <5 years of age listing the International Classification of Diseases, 9th Revision, Clinical Modification (ICD-9-CM) code for MC (078.0) [20].

The outpatient visit records for AI/AN children <5 years of age with a MC diagnosis were examined by sex, age group (<1 and 1–4 years) and IHS region. The IHS West region, a region with a high incidence of MC [15], was further analyzed by Service Unit to identify those Service Units with the highest MC incidence. Annual and average annual MC-associated incidence rates per 10,000 children <5 years of age from the corresponding group were calculated for the study period using the annual number of patients with a MC diagnosis and the corresponding annual fiscal year IHS user population as the denominator. The IHS user population includes all registered AI/AN persons who have received IHS-funded health care services at least once during the preceding three years [19].

### Case-control study

A case-control study was conducted to identify clinical characteristics of dermatological conditions among children <5 years of age that may be associated with the occurrence of MC using chart review at Facility B. Facility B is contained within Service Unit B, a service unit with a high annual incidence of MC in children <5 years of age (Table 1). The MC outpatient cases were identified as described below. Controls were randomly selected as children <5 years of age with outpatient visits during the same time period as cases, and did not list a current or prior MC diagnosis. Complete medical charts were abstracted for 84 cases and 109 controls at Facility B. Data from MC case patients were abstracted if the specific diagnosis of MC was listed on the medical chart. The specific dermatological conditions abstracted from medical charts included candidiasis; dermatitis; dry skin; eczema; hand, foot, and mouth disease; impetigo; rash; ringworm; scabies; varicella; viral exanthem; and viral warts. The diagnoses for additional conditions were captured as either co-occurring with the MC diagnosis and/or occurring prior to the MC diagnosis. A univariate analysis was conducted to identify clinical characteristics associated with MC. We refer to these significant characteristics as ‘risk factors’ noting that this analysis does not identify causation, but rather an association of a characteristic with MC.

**Table 1:** Incidence rates^a^ of molluscum contagiosum in American Indian children <5 years of age, fiscal years 2001–2009.

	Overall	Sex		Age (years)	
		Male	Female	<1	1–4
Service Unit A	188.4	179.2	198.7	45.1	212.7
Service Unit B	222.8	198.5	247.7	31.9	265.3
IHS Overall	68.5	67.4	69.6	19.2	77.6
Region					
Alaska	83.2	77.7	89.1	24.4	96.6
East	121.5	132.5	119.4	40.5	135.3
Northern Plains East	77.8	75.0	80.7	19.4	87.1
Northern Plains West	71.4	67.0	75.8	19.9	81.0
Southern Plains	51.1	53.2	48.9	16.5	56.7
Southwest	48.3	48.4	48.2	13.3	55.2
West	132.9	129.6	136.4	35.1	148.2

a Incidence rates are reported as per 10,000 children <5 years of age.

### Characteristics of MC cases at two outpatient clinics

Two IHS facilities (Facilities A and B) in the northwest United States were identified from IHS Service Units A and B, respectively, with a high incidence of MC among AI/AN children <5 years of age (Table 1). A chart review of records was conducted for all MC-associated outpatients <5 years of age during fiscal years 2001–2009 at the two facilities. A data collection form was developed to extract demographic and clinical information from medical records for these patients.

The ICD-9-CM code diagnosis of MC was validated by a healthcare provider's noted diagnosis entered on the medical record, and patients meeting these criteria were considered MC cases. Data were abstracted from a total of 175 cases (97.2% of identified ICD-9-CM diagnoses) from Facility A and 84 cases (81.6% of identified ICD-9-CM diagnoses) from Facility B. Data were not abstracted from all of the ICD-9-CM diagnosed cases because of missing medical records (n = 24, 8.5%) or misdiagnoses (n = 8, 2.8%). Age in months at first visit was compared using the Wilcoxon rank-sum test [21]. Statistical significance was considered at the p<0.05 level for all analyses.

### Ethics Statement

The chart review at the two health facilities was determined to be public health non-research by CDC and exempt from review by the Portland Area IHS Human Subjects Institutional Review Board. Patient data was de-identified and anonymized prior to analysis.

## Results

### Incidence of MC at IHS outpatient facilities nationwide

The incidence of MC infections was calculated from IHS outpatient visit records for AI/AN children <5 years of age (Table 1). The overall incidence rate was 68.5 per 10,000 children, and the rate was similar amongst males and females. Children aged 1–4 years had an incidence rate four times higher than the rate for children <1 year old. The incidence rate was highest in the West (132.9) and East (121.5) IHS regions. Service Units A and B (including corresponding Facilities A and B) had particularly high incidence rates of 188.4 and 222.8, respectively. At Service Unit B, the incidence rate for children aged 1–4 years old was eight times that of children <1 year old.

### Dermatological risk factors for MCV infection

Patient charts were reviewed from 175 cases (67.6%) at Facility A and 84 cases (32.4%) at Facility B. There were slightly more female (52.1%) than male patients (47.9%) (Table 2). The average age of patients at their first diagnosis of MC was 35 months with a median of 36 months. Approximately 95% of MC patients were one year of age or older (Table 2).

**Table 2:** Demographic characteristics of American Indian molluscum contagiosum case and control patients <5 years old by facility.

	Facility A	Facility B	
	Cases^a^	Cases^a^	Controls^a^
Total	175 (100)	84 (100)	109 (100)
Sex			
Male	87 (50)	37 (44)	50 (46)
Female	88 (50)	47 (56)	59 (54)
Age (years)			
<1	9 (5)	5 (6)	40 (37)
1–4	166 (95)	79 (94)	69 (63)

a Number of patients (%).

Data were collected for each visit where MC was diagnosed, including multiple visits. The majority of cases (68.7%) had only one diagnosis of MC. However, a substantial proportion of patients had multiple diagnoses over time: 19% of patients had a MC diagnosis for two visits, 9.7% for three visits, 1.9% for four visits, and 0.8% (2 patients) for five visits. Common descriptions for lesion presentation included characteristic ‘pitted’ or ‘umbilicated’ lesions (49.3%), ‘colorless’ (44.8%), ‘red’ or ‘pink’ (17.2%), erythema (16.4%), and pruritus (25.4%).

A case-control study to evaluate potential dermatological risk factors for MCV infection was conducted using medical records obtained at Facility B. The gender distribution was similar among MC cases and controls, and controls were significantly younger than case patients (p<0.0001, Table 2). Control patients presented to the clinic with a variety of chief complaints, whereas the chief compliant for most of the visits for case patients was self-described bumps or rash (84.3%).

Dermatological conditions were examined as potential risk factors for MCV infection at three different time periods relative to the visit - a) prior dermatological conditions, b) co-occurring dermatological conditions, and c) a combination of prior and co-occurring conditions. Considering prior dermatological conditions only, cases were more likely to have had a prior diagnosis of eczema (OR = 2.51, 95% CI 1.10–6.01), eczema or dermatitis (OR = 2.09, 95% CI 1.16–3.81), impetigo (OR = 2.88, 95% CI 1.40–6.10), or scabies (OR = 3.72, 95% CI 1.04–17.40) compared to controls (p<0.05) (Table 3). Considering co-occurring conditions only, cases were more likely to have diagnoses of eczema (OR = 5.71, 95% CI 1.98–20.66), eczema or dermatitis (OR = 2.97, 95% CI 1.24–7.69), or impetigo (OR = 4.86, 95% CI 1.14–33.24) than controls (p<0.05) (Table S1 in File S1). Grouping past and co-occurring conditions together, eczema (OR = 3.58, 95% CI 1.77–7.52), eczema or dermatitis (OR = 2.28, 95% CI 1.28–4.11), impetigo (OR = 3.40, 95% CI 1.73–6.92), and scabies (OR = 3.72, 95% CI 1.04–17.40) were more likely to occur in MC cases compared to controls (p<0.05) (Table S2 in File S1).

**Table 3:** Univariate analysis of prior dermatological conditions for American Indian molluscum contagiosum (MC) cases and control patients <5 years of age at facility B.^a^

Condition	Case N (%)	Control N (%)	OR (95% CI)	p-value
Eczema				
No	67 (79.8)	99 (90.8)	Reference	
Yes	17 (20.2)	10 (9.2)	2.51 (1.10–6.01)	0.029
Eczema or Dermatitis^b^				
No	44 (52.4)	76 (69.7)	Reference	
Yes	40 (47.6)	33 (30.3)	2.09 (1.16–3.81)	0.014
Impetigo				
No	59 (70.2)	95 (87.2)	Reference	
Yes	25 (29.8)	14 (12.8)	2.88 (1.40–6.10)	0.004
Scabies				
No	76 (90.5)	106 (97.2)	Reference	
Yes	8 (9.5)	3 (2.8)	3.72 (1.04–17.40)	0.043

^a^Significant variables (conditions) are shown here. Non-significant variables were candidiasis; dermatitis; dry skin; hand, foot, and mouth disease; rash; ringworm; varicella; viral exanthem; and viral warts.^b^This variable includes diagnoses of eczema or dermatitis.doi:10.1371/journal.pone.0103419.t003

**Figure 1:**
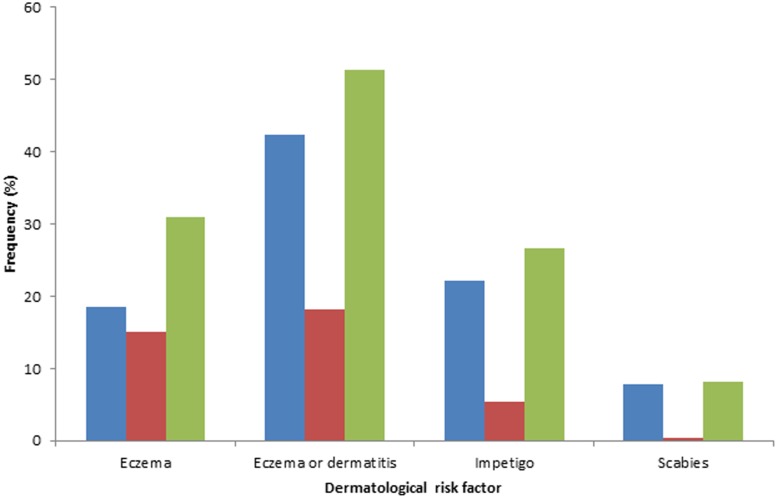
The frequency of dermatological risk factors in MC case patients at three time periods relative to the initial visit where MC was diagnosed - a) prior dermatological conditions (blue), b) co-occurring dermatological conditions (red), and c) a combination of prior and co-occurring conditions (green). MC cases include those from Facilities A and B.

The frequencies of these risk factors in MC patients at both facilities and for the three time periods examined are shown in Figure 1. In considering any time period, 30.9% of MC cases had a diagnosis of eczema and 51.4% had a diagnosis of eczema or dermatitis. Also, 26.6% and 8.1% of MC cases had a diagnosis of impetigo or scabies, respectively.

## Discussion

Published data show a higher incidence of MC in AI/AN children <5 years of age, specifically amongst children aged 1–4 years [15]; and the more recent measures here show the same trend across IHS regions, with the West region having the highest incidence. Moreover, this age distribution has been noted previously in children <5 years old [13], [22]–[24]. Some authors have hypothesized that children in this age range have behaviors and activities that facilitate close contact with affected individuals, thereby facilitating the spread of MCV to others [23], [25], and others have suggested that maternal antibodies may protect newborn infants from infection [23]. Cases from the two IHS facilities examined in the present study had a slight predominance of female patients versus male. There are no consistent data in the literature to suggest one gender is overrepresented by MCV infections [8].

The majority of MC cases in the present study were noted to have a MC diagnosis only once, but over 30% of cases had MC on multiple healthcare center visit records. The duration of illness lasted for several months for many patients and up to one year for one patient. MC is known to persist for months to years, eventually resolving without treatment for many patients [3].

In this population of pediatric AI/AN patients with a high burden of MC, there was an association between MC and prior or co-occurring diagnosis of eczema, eczema or dermatitis, impetigo, and scabies. Prior research has suggested a purported association between patients with AD or eczema and MC [25]–[27], but this study is the first to quantify this relationship. AD patients are known to have an increased susceptibility to many dermatologic infections and are at risk for severe complications from poxviral infections, notably *Orthopoxviruses* including vaccinia virus [22], [28], [29]. A study that utilized parent recall of prior dermatological conditions in pediatric patients did not find an association between MC illness and prior history of AD [30], but this approach has the limitation of dependence on recall of clinical diagnosis. Here, we utilized documented clinical diagnoses of MC and concurrent or prior diagnosis of several dermatological skin conditions. All of the significant conditions (eczema, eczema or AD, impetigo, scabies) can compromise the integrity of the external epidermal surface, providing portals of entry for the establishment of viral infections. Eczema and AD can also be considered manifestations of immune deficits or disregulation, and this may yield a propensity to develop infections [22].

A recent study of pediatric health survey data suggested that approximately 10% of all children <17 years old in the United States have had a prior diagnosis of eczema, and children <4 years old have the highest incidence of eczema. Also, the incidence of eczema differed geographically, with the western United States having a notably high incidence (although, high incidence rates were is not observed exclusively in the western United States) [31]. Over half of the pediatric MC patients in the present study had a diagnosis of eczema or dermatitis, a remarkably high frequency of diagnoses compared to national data. In a published chart review by Berger, et al., a high percentage of pediatric MC patients (37.2%) had a history of AD [28]. It is unknown if the co-occurring high incidence of eczema and MC are related.

The present study has several possible limitations, as well as, some unique attributes that contribute to the strength of our conclusions. Because the AI/AN populations studied are relatively isolated, the IHS clinic, which offers no-cost health care to eligible beneficiaries, is the source of the vast majority of health care for these populations. It is believed that the charts at Facilities A and B represented most of the clinical visits for an individual over their life to date (up to 5 years of age), so our ascertainment of prior dermatological conditions was likely to be complete. Also, this study was able to capture and review the majority of MC cases <5 years of age at the two outpatient clinics. However, clinical diagnoses (including those for impetigo and scabies) were based on clinical examination alone and never laboratory confirmed, which likely suggests that some diagnoses may have been inaccurate. Diagnostic laboratory testing was only requested for one MC case and the results were not listed in the chart. Similarly, in most diagnoses of AD there was no documentation of any additional laboratory evaluation or referral to a dermatologist for evaluation. Further, surrounding erythema is known to occur with some MC lesions and this was noted for 16.4% of MC cases reviewed here. It is unknown if the presence of erythema surrounding lesions was considered by the practitioners to be a presentation of MC or a presentation of atopy. Such possible misdiagnoses could lead to a misclassification bias in our study, although unless primary care providers systematically misdiagnosed MC for AD, there should not be a significant effect on our measures of association. Further, descriptive information was missing from some charts, particularly information relevant to the MC presentation.

The AI/AN population harbors a high burden of infectious diseases [32], [33], and MC in the pediatric population is no exception. The present study affirms the association between AD and MC; however, the mechanisms of such an association are unknown and deserve further attention and study in additional patient populations. AI/AN pediatric populations would benefit from targeted outreach to prevent and mitigate the spread of MCV among children.

## Supporting Information

File S1 **Supporting tables.** Table S1, Univariate analysis of co-occurring dermatological conditions for American Indian molluscum contagiosum (MC) cases and control patients <5 years of age at facility B. Table S2, Univariate analysis of previous and current dermatological conditions for American Indian molluscum contagiosum (MC) cases and control patients <5 years of age at facility B.
(DOCX)
