# Supplementary material for: Molluscum Contagiosum in a Pediatric American Indian Population: Incidence and Risk Factors
Source: PLoS One. 2014 Jul 29;9(7):e103419. doi: 10.1371/journal.pone.0103419 (PMC4114779; doi:10.1371/journal.pone.0103419)
Supplement: File S1 — Supporting tables. Table S1, Univariate analysis of co-occurring dermatological conditions for American Indian molluscum contagiosum (MC) cases and control patients <5 years of age at facility B. Table S2, Univariate analysis of previous and current dermatological conditions for American Indian molluscum contagiosum (MC) cases and control patients <5 years of age at facility B. (DOCX) [file pone.0103419.s001.docx]

Table S1. Univariate analysis of co-occurring dermatological conditions for American Indian molluscum contagiosum (MC) cases and control patients < 5 years of age at facility B.^a^

| Condition | Case N (%) | Control N (%) | OR (95% CI) | p-value |
| --- | --- | --- | --- | --- |
| Eczema |  |  |  |  |
| No | 69 (82.1) | 105 (96.3) | Reference |  |
| Yes | 15 (17.9) | 4 (3.7) | 5.71 (1.98 – 20.66) | 0.001 |
| Eczema or Dermatitis^b^ |  |  |  |  |
| No | 68 (81.0) | 101 (92.7) | Reference |  |
| Yes | 16 (19.0) | 8 (7.3 ) | 2.97 (1.24 - 7.69) | 0.015 |
| Impetigo |  |  |  |  |
| No | 77 (91.7) | 107 (98.2) | Reference |  |
| Yes | 7 (8.3) | 2 (1.8) | 4.86 (1.14 – 33.24) | 0.032 |

^a^Significant variables (conditions) are shown here. Non-significant variables were candidiasis; dermatitis; dry skin; hand, foot, and mouth disease; rash; ringworm; scabies; varicella; viral exanthem; and viral warts.

^b^This variable includes diagnoses of eczema or dermatitis.

Table S2. Univariate analysis of previous and current dermatological conditions for American Indian molluscum contagiosum (MC) cases and control patients < 5 years of age at facility B.^a^

| Condition | Case N (%) | Control N (%) | OR (95% CI) | p-value |
| --- | --- | --- | --- | --- |
| Eczema |  |  |  |  |
| No | 55 (65.5) | 95 (87.2) | Reference |  |
| Yes | 29 (34.5) | 14 (12.8) | 3.58 (1.77 - 7.52) | 0.000 |
| Eczema or Dermatitis^b^ |  |  |  |  |
| No | 37 (44.0) | 70 (64.2) | Reference |  |
| Yes | 47 (56.0) | 39 (35.8) | 2.28 (1.28 - 4.11) | 0.005 |
| Impetigo |  |  |  |  |
| No | 53 (63.1) | 93 (85.3) | Reference |  |
| Yes | 31 (36.9) | 16 (14.7) | 3.40 (1.73 – 6.92) | 0.000 |
| Scabies |  |  |  |  |
| No | 76 (90.5) | 106 (97.2) | Reference |  |
| Yes | 8 (9.5) | 3 (2.8) | 3.72 (1.04 – 17.40) | 0.043 |
|  |  |  |  |  |

^a^Significant variables (conditions) are shown here. Non-significant variables were candidiasis; dermatitis; dry skin; hand, foot, and mouth disease; rash; ringworm; varicella; viral exanthem; and viral warts.

^b^This variable includes diagnoses of eczema or dermatitis.
